# AI-Assisted Decision-making in Healthcare: The Application of an Ethics Framework for Big Data in Health and Research

**DOI:** 10.1007/s41649-019-00096-0

**Published:** 2019-09-12

**Authors:** Tamra Lysaght, Hannah Yeefen Lim, Vicki Xafis, Kee Yuan Ngiam

**Affiliations:** 1 Centre for Biomedical Ethics, Yong Loo Lin School of Medicine, National University of Singapore, Singapore; 2 Nanyang Business School, Nanyang Technology University, Singapore; 3 Division of General Surgery (Thyroid & Endocrine Surgery), Department of Surgery, National University Hospital, Singapore

**Keywords:** Artificial intelligence, Big data, Clinical decision-making support systems, Professional governance, Bioethics

## Abstract

Artificial intelligence (AI) is set to transform healthcare. Key ethical issues to emerge with this transformation encompass the accountability and transparency of the decisions made by AI-based systems, the potential for group harms arising from algorithmic bias and the professional roles and integrity of clinicians. These concerns must be balanced against the imperatives of generating public benefit with more efficient healthcare systems from the vastly higher and accurate computational power of AI. In weighing up these issues, this paper applies the deliberative balancing approach of the *Ethics Framework for Big Data in Health and Research *(Xafis et al. 2019). The analysis applies relevant values identified from the framework to demonstrate how decision-makers can draw on them to develop and implement AI-assisted support systems into healthcare and clinical practice ethically and responsibly. Please refer to Xafis et al. (2019) in this special issue of the Asian Bioethics Review for more information on how this framework is to be used, including a full explanation of the key values involved and the balancing approach used in the case study at the end of this paper.

## Background

Artificial intelligence (AI) is playing an increasingly important role in healthcare. AI software platforms are currently being developed or implemented for use in many targeted healthcare applications, including (but not limited to) medical diagnostics, patient monitoring, and learning healthcare systems. This domain focuses on AI algorithms and software being developed to support clinical decision-making and/or public health policymaking. These AI algorithms typically employ computerised predictive analysis algorithms to filter, organise, and search for patterns in big data sets from multiple sources and provide a probability analysis upon which healthcare providers can make fast and informed decisions. At present, most jurisdictions do not allow these algorithms to be the final decision-maker. Instead, they are mostly used as a screening tool or as an aid to diagnosis.

In the past few years, AI-assisted data analysis and learning tools have been used widely in research with patient electronic health records (EHRs). These records were previously kept on paper within hospitals but now exist electronically on secure computer servers. The increasing computational power of hardware and AI algorithms is also enabling the introduction of platforms that can link EHRs with other sources of data, such as biomedical research databases, genome sequencing databanks, pathology laboratories, insurance claims, and pharmacovigilance surveillance systems, as well as data collected from mobile Internet of Things (IoT) devices such as heart rate monitors. AI-assisted analysis of all this big data can generate clinically relevant information in real time for health professionals, health systems administrators, and policymakers. These *Clinical Decision Support Systems* (CDSS) are programmed with rule-based systems, fuzzy logic, artificial neural networks, Bayesian networks, as well as general machine-learning techniques (Wagholikar et al. 2012). CDSS with learning algorithms are currently under development to *assist* clinicians with their decision-making based on prior successful diagnoses, treatment, and prognostication.^1^

This domain focuses on the ethical and governance challenges arising from the development and implementation of AI-assisted CDSS within clinical- or practice-based contexts and research. Generally speaking, research encompasses knowledge-generating activities while clinical practice focuses primarily on patient care. Even though similarities exist in the ethical norms that govern both activities (e.g. obtaining informed consent and maintaining confidentiality), there are also differences in how risks and benefits are weighed up. In clinical contexts, known and potential harms are balanced against the benefits that are expected for the individual patient. In research, studies are not normally designed to offer individual participants tangible benefits. Rather, the risks of harms to participants must be justified by the public benefits of producing generalisable knowledge that can improve clinical practice and healthcare for future patients. Similarly, patients whose data was collected many years ago and fed into the system would have fewer benefits than those who presently require clinical care that can benefit from the AI-assisted decision-making capacity. Thus, ethical evaluation of AI-assisted CDSS must carefully consider and balance the differing interests and values.

In this domain, we draw on three examples to illustrate how these issues can emerge:

1. where clinicians use AI-assisted CDSS to provide diagnoses to patients and predict treatment outcomes based on relevant clinical data, such as medical and social history, socio-demographics, diagnostic tests, and genome sequences, that are recorded into patient’s EHR and fed back into the CDSS for incremental machine learning;
2. where AI-assisted CDSS is used to conduct research on patient populations to calculate the risks of non-compliance to prescribed management plans for individual patients; and
3. where the AI-assisted CDSS is used to generate knowledge bases to improve system-wide efficiencies and patient outcomes in learning healthcare systems.

Our analysis suggests it is important that developers and implementers of AI-assisted CDSS put in place control mechanisms to protect individuals and groups from harms arising through the tools. At the same time, these controls must not be so restrictive as to prevent the public benefits they can also deliver for health systems. For the purposes of this domain, ‘developers’ are the organisations and individuals who develop and maintain CDSS software solutions or applications that make use of AI technology’; and ‘implementers’ as the organisations and individuals who use AI technology to deliver a healthcare service. Implementers can include hospitals and healthcare professionals who incorporate AI-assisted CDSS into the provision of healthcare services, as well as researchers who collect the data that is input into the CDSS and test the efficacy of the AI-algorithms. An organisation and/or individual can be both developers and implementers of these systems.

## Key issues

There is already substantial literature on CDSS and ethics (e.g. Goodman 2007). However, very little of the existing literature deals with the deep and complex functionalities and issues surrounding AI-driven systems, which are by and large not run on hard-coded software and pre-vetted data^2^ like the systems of past. Further, existing literature on CDSS and ethics largely deal with CDSS which are often patented so that the methodology is open for public scrutiny. However, the nature of machine learning means that the large data sets on which the tools are trained on may not be made available for public for scrutiny. By its very nature, AI-driven systems are unique and new in how they function when compared with other types of CDSS that may have been in existence for decades; hence, they present issues more acute than those of previous types of CDSS. The four issues we consider here are the following:

1. Accountability and transparency of the decisions AI-assisted systems make
2. The potential for group harms arising from biases built into AI algorithms
3. Public interest in generating more efficient healthcare from AI-assisted systems
4. The potential conflicting professional roles and duties of clinicians as both users and generators of research data for AI-assisted systems

### Example 1: CDSS for Assisting with Diagnoses

**Table:** 

Hospitals and healthcare systems are introducing CDSS platforms to enable the use of machine learning to assist with diagnostic decisions and to predict treatment outcomes. The CDSS works by continuously monitoring information that clinicians enter into the EHR. As information is recorded, the CDSS can analyse the entries in real time along with other clinically relevant data that is linked to the EHR from other unrelated sources. These sources may include test results from pathology laboratories, radiological departments, genetics departments, and ambulatory settings, as well as research results stored in biobanks, clinical trials, and databanks of genome sequences. The CDSS can then make diagnostic recommendations based on algorithms that are typically programmed using rules informed by established clinical guidelines and published medical research reviews. Many diagnostic applications have been proposed for CDSS and some programs been approved for marketing as medical devices in several countries. This includes software with deep-learning algorithms that can analyse medical images to diagnose cardiovascular disease, wrist fractures, stroke, and diabetic retinopathy. Other applications undergoing clinical trials include programs use to diagnose breast and skin cancers, congenital cataract disease, Parkinson’s disease, and diabetes mellitus (Jiang et al. 2017). In many instances, the machine learning has been demonstrated to be at least as accurate as an experienced clinician with the added advantage of being able to recommend a diagnosis much faster. As the diseases being targeted for CDSS are typically chronic and/or degenerative, early diagnoses are critical for reducing complications, controlling symptoms, and improving outcomes. For example, CDSS can analyse the data stored in the EHR on patients already diagnosed with diabetes, such as symptoms, medical history, physical examinations, lab tests, treatments, and outcomes to make predictions and to formulate a possible diagnosis for a patient with similar traits and characteristics (El-Sappagh and Elmogy 2016). Similarly, the software is able to predict the likely outcomes of treatment options by accounting for information that a clinician may not readily have access to or be unaware of when recommending a management plan to their patient. The platforms can alert clinicians to potential problems and conflicts with the treatment plans, such as likely drug-efficacy probabilities, and allow the doctor to revise the management plan accordingly.	

### Accountability and Transparency

One of the major concerns about AI-assisted CDSS is how the machines reach decisions, and whose decision should prevail when there is disagreement between the CDSS and the medical professional. Unlike traditional software, the machine learning algorithms and the models AI uses to make decisions are highly complex and, in some cases, opaque. While methods are available to allow users to visualise the factors that the machine has learned in making a particular prediction (e.g. graphical-based networks and attention mechanisms), some techniques are indecipherable even to software engineers (Burrell 2016). This lack of transparency is referred to as the ‘black box’ of AI. In addition to the lack of transparency, the necessary use of large training data sets coupled with mathematical and statistical algorithms and sometimes neural networks, whether with or without full understanding of the internal workings, presents a challenge in educating doctors to use these tools in a clinically relevant way.

For the CDSS in Example 1, it is imperative that these tools are designed to be explainable in order for doctors to understand how the machine has arrived at its recommended diagnosis or has flagged out an alert. It is crucial that these tools are trained based on validated data sets using methods that have undergone a peer-review process. Based on published data on the performance characteristics of the tool, it would allow the clinician to understand how the algorithm has assigned weights to the data. Based on this understanding, the clinician can then determine whether she should override the recommendations given by the CDSS or not. In this scenario, it is entirely acceptable for a clinician to use the factors and suggestions of a validated AI CDSS to *assist* them to make better decisions, based on published sensitivities and specificities of that AI tool. This is analogous to a doctor using an X-ray machine to aid in the diagnosis of a patient—doctors are not expected to know the intricate engineering of an X-ray machine, but must know the performance characteristics and limitations of the machine that are necessary to help them arrive at a diagnosis.

Given that medical practitioners have an ethical and legal duty of care to their patients and are responsible for the clinical recommendations and decision-making, transparency of how their decisions are made, with or without an AI-assisted CDSS, needs to be clear. However, it remains to be seen if an AI algorithm could be held to meet the required standard of care under negligence law. For example, in Singapore, the Court of Appeal in *Hii Chii Kok v Ooi Peng Jin London Lucien and another* [2017] SGCA 38 has distinguished the relevant duties and standard of care required of a doctor at the diagnosis, advice and treatment stages and set a different standard of care required at the advice stage. This decision has ramifications for how and at which stage an AI-assisted CDSS is used.

Given this legal framework, if a patient is harmed as a result of a doctor’s decision to override a CDSS recommendation, then the doctor’s decision-making against the AI algorithm will come under scrutiny. This situation could arise where the AI algorithm accounts for information about a patient’s symptoms that the practitioner could reasonably be expected to know, but she or he neglects the information and misdiagnoses the patient’s condition. Having access to the AI system’s weighted factors will be crucial to account for these decisions, and to determine if something was overlooked or contentious. In such cases, a patient would have to show that they lost the chance of a better outcome as a result of the doctor’s failure to interpret the AI tool’s recommendation. These kind of judgements are presently untested and might be avoided if the role of AI tools is maintained as decision support tools subject to final interpretation by doctors.

As AI algorithms develop and become more ubiquitous in the future, there are concerns about doctors becoming over-reliant on AI assistance and complacent in their decision-making. These concerns will likely depend on the types of decisions the CDSS is assisting the doctor with. For example, diagnostics are primarily based on statistical prediction and there is increasing evidence suggesting that AI is as good at calculating these probabilities as humans whereas, prescribing and treatment options involve careful discussions with patients to align the chosen management plan with their values, goals, and preferences. Humans are far better at engaging in these personal discussions and making value judgments than AI, which is so far unable to detect contextual factors and social cues.

Indeed, AI may allow doctors to spend more time having personal discussions with patients while leaving time-consuming statistical calculations and predictions to the CDSS. Spending more time to provide better care enhances patient trust, which is foundational to the relationship between medical practitioners and patients. However, practitioners also need to take care that the AI-assisted CDSS does not obstruct the patient-doctor relationship and they must realise that the legal and moral responsibility for decisions made rests with them. Thus, implementers may need to ensure that doctors are adequately trained on the benefits and pitfalls of AI-assisted CDSS and apply them in practice to *augment* rather than replace their clinical decision-making capabilities and duties to patients.

A final point to make about transparency is the degree of information that should be provided to patients about the limitations of the AI systems that are assisting clinicians with their decision-making. Classic formulations of informed consent require disclosure of materially relevant information. Material information is that which an individual would consider important and likely to influence their decision; for example, information required to accept a diagnosis and consent to a proposed management plan. How much information about the AI’s algorithms, its limitations, and influence over the doctors’ decisions would, or should, be considered ‘material’ is unclear.

#### Example 2: CDSS for Risk Calculation

**Table:** 

As suggested in the example above, CDSS can be used to predict patient outcomes. These predictions are not only clinically useful: they can also be used to help inform other interested parties which groups of patients are likely to have better outcomes. Public and private health insurers are parties that would benefit from access to this information. Knowledge acquired by AI may be used to build profiles of patients based on aggregated data in the EHR and characteristics, such as genetic traits, lifestyle preferences, and socio-demographics. Public health systems might use this information to predict which patients are likely to require re-hospitalisation and prioritise resources accordingly. Private health insurers could also associate these profiles with levels of risk to calculate insurance premiums and/or offer customised packages for disease groups not covered under existing insurance plans. Going back to the previous example, some insurers, for example, may not cover patients for complications arising from diabetes mellitus (see, for, e.g. in the USA: Guo et al. 2017). Possible complications of diabetes can be wide ranging and include heart disease, kidney disease, retinopathy, foot ulceration, and skin conditions. Patients who are diagnosed early and have profiles that fit one likely to comply with prescribed management plan, make lifestyle adjustments, and avoid further complications, as predicted by the AI system, might still be eligible for insurance coverage under plans tailored for this risk group. However, patients who fit the profile of high-risk groups unlikely to have good outcomes may remain under-insured or be forced to pay higher premiums for coverage of certain complications associated with the disease.	

### Risks of Group Harms

Another major concern about the AI algorithms of the CDSS is the potential for the system to reinforce and potentially exacerbate social prejudices and biases that can harm certain groups and communities. Example 2 indicates how this concern might emerge even with strong data protections and security in place. AI-assisted CDSS can be programmed to detect correlations and associations between certain diagnoses and a large range of genetic and social factors that may include gender, ethnicity, disability, socio-economic background, education, employment status, and geographical living space. The CDSS can predict outcomes based on these and other social determinants of health, which can lead to further discrimination of already-marginalised groups and communities, as discussed in Xafis et al. (2019).

One particularly powerful social determinant of health is the ability to access healthcare (McGibbon et al. 2008; National Academies of Science 2017). There is a potential for CDSS to further impact on those less able to afford healthcare services. Individual and group level harms can arise if information drawn from the CDSS can be used to determine the level of insurance coverage patients can receive based on social characteristics. For example, patients from disadvantaged groups, that have fewer economic and social resources to access healthcare, tend to have poorer health outcomes. Conversely, individuals from advantaged groups with better insurance coverage are likely to continue doing well economically and socially because good health promotes these opportunities. If individuals within the disadvantaged groups are denied health insurance or receive only limited coverage, then discriminatory harms may be perpetuated (Guo et al. 2017; Rosenbaum 2009).

While some jurisdictions, such as the USA (Rosenbaum 2009), have legislation in place to protect populations from this type of discrimination, not all do (for example, Singapore does not) and even where it does exist, the law may not prevent private health insurers from tailoring plans based on information generated from the CDSS.^3^ Many jurisdictions have introduced legislation that prohibits the sharing of personally identifiable data in the EHR without informed consent from patients.^4^ However, these laws often do not include de-identified data sources and would not prevent third parties accessing aggregated information in the CDSS for risk profiling.^5^ They also would not prevent any stigmatisation that may arise from certain diseases being associated with marginalised groups.

#### Example 3: CDSS for Learning Health Systems

**Table:** 

In addition to providing information relating to clinical care of individual patients, CDSS may also be used for population level learning to generate knowledge bases in what are known as ‘learning healthcare systems’ (LHS). LHS are systems in which processes for generating knowledge through comparative effectiveness research and quality improvement programs are embedded into routine practice to continually improve healthcare service delivery and patient outcomes (Institute of Medicine 2007). Within these systems, CDSS platforms are able to bring together many diverse and rich data streams, which include the EHR, as well as laboratory information systems, data repositories, administrative claims data, patient registries, and post-marketing surveillance (Yu 2015). These sources of dynamic and live real-world data generate practice-based evidence that can supplement evidence gathered from the basic sciences and clinical trials. While health system research already generates this type of knowledge base, machine learning analytics in CDSS can vastly speed up the process to deliver better and more efficient healthcare services, in as close to real time as possible. These platforms can more effectively close the loop between the care a patient is given, as documented in the EHR and administrative data, and the healthcare provider. An example is the MOSIAC platform, developed in the EU, with the aim of supporting diabetes management. This platform integrates diverse data sets from hospitals and public health repositories that are exploited using advanced temporal analytics tools that focus on diabetes complications (Dagliati et al. 2018b). Information generated in this system can be used to deepen the insights on diabetes monitoring, allow better understandings of clinical dimensions, recognise novel phenotypes, inform clinical actions, and improve the efficiency of the health system (Dagliati et al. 2018a).	

### Balancing Individual and Group Harms with Public Interests in Efficient Health Systems

Besides the possible benefits to individual patients, AI-assisted CDSS may also deliver social benefits with more efficient public health systems. As the costs of healthcare continue to rise, it is an imperative for policymakers to identify and implement more cost-effective measures and to remove inefficiencies that place unnecessary burdens on public health spending. Maintaining sustainable health systems is thus an important public value that must be balanced against the need to protect and minimise the potential harms from the misuse of AI to individuals and groups.

Example 3 indicates how AI-assisted CDSS might deliver those benefits within a LHS. Hospital administrators can, for example, adopt knowledge generated from the CDSS about the cost-effectiveness of a generic drug over an expensive branded one, or whether a particular operational process saves on time and labour. While administrators are already able to undertake these analyses, the processing power of AI to sort through the large number of variables in the health system and identify patterns can accelerate this process. The faster this information is known, the more quickly the hospital’s purchasing policies and standard operating procedures can adapt, saving money and resources that can be allocated elsewhere in the system. However, realising these benefits will require free and rapid flow of information from the EHR to the CDSS platform and into reportable outputs that can be validated and disseminated to others outside the doctor-patient relationship. This will require some trade-offs with the control that patients have over the information that is contained in the EHR and public health imperatives. To circumvent this, the researchers and administrators could use aggregated, de-identified data to undertake their analysis. However, it must be noted that no data can be truly de-identified (Lim 2017).

### Conflicting Professional Roles and Duties of Clinicians

An underlying issue in all three examples above is the blurring line between the use of AI-assisted CDSS for clinical practice and research.^6^ These systems are programmed to continuously include and analyse data as patient care is documented in the EHR and other sources of information become available. The insights from this process can be tested and validated, and ultimately contribute to a knowledge base that will inform future practice; both within the healthcare institution of the patient and more widely with the revision of clinical guidelines and health policies. Although most AI-assisted CDSS systems are still in research stages, as they become accepted into clinical practice, their capacity to perform ongoing research tasks will likely become less distinguishable as separate functions.

One ethical implication of this blurring boundary (Kass et al. 2013) is the dual-role that doctors will increasingly play as both clinical practitioners and researchers when interacting with the CDSS. As practitioners, the primary concern of doctors is patient care. They document clinical observations about patients either directly into the EHR or as unstructured narrative text (i.e. dictated and transcribed). This information can be standardised for the CDSS using natural language processing methods that AI algorithms can adapt to. However, the system is more accurate when doctors enter this information in a structured way; more so than they might do otherwise if the primary concern is patient care. The more systematic doctors are at entering information into the EHR—like how a researcher would record and document their observations—the higher the quality of the overall dataset, and the more reliable the recommendations of the CDSS are.

In this scenario, the clinician is no longer solely concerned with recording information about patient care but that it is documented in ways that are useful both for clinical practice and for research purposes. Potential conflicts in the dual-role may arise when doctors feel their obligation to dutifully record information in the EHR for research purposes obstructs their professional obligations to patient well-being (Goodman 2010). These conflicts might be reduced if AI systems are integrated into clinical care so that doctors do not have to disrupt their work to key in duplicate or additional data for research purposes.

## Relevant Values

In the section, we identify values from the Ethics Framework for Big Data (Xafis et al. 2019) for developers and implementers of AI-assisted CDSS to consider in light of the issues discussed above. They are not the only relevant values but we consider them the most salient ones that can come into conflict and may be usefully balanced in ethical decision-making for developing and implementing AI-assisted CDSS into healthcare settings.

### Substantive Values

#### (Professional) Integrity

Maintaining professional integrity when interacting with the AI-assisted CDSS and incorporating it into practice. This will involve implementing systems that encourage doctors to interact with AI-assisted CDSS in ways that *augment* their clinical decision-making without compromising their primary responsibilities and duties to patient care. At this present time, developers should program the AI algorithms to ensure that a qualified healthcare professional remains the final decision-maker irrespective of the recommendations of the CDSS and that implementers are made aware when decisions will require moral judgements from the doctors and their patients. Implementers need to ensure adequate training is provided to healthcare professionals using the software to avoid any over-reliance or complacency, and recognise that the AI-algorithms are unable to make moral judgements.

#### Justice

Adhering to considerations of justice by recognising that AI-assisted CDSS may replicate social biases and prejudices that can bring about discriminatory harms and ensuring that the benefits and burdens of these systems are distributed equitably across the population, developers must be aware of any existing biases in the training data sets that have been used. Where there are risks of the AI system learning and replicating social biases and prejudices that can disadvantage certain populations, developers should be transparent about such shortcomings and have built in indicators to detect problems as they emerge, and report them back to the developer for further development. To optimise this feedback, frameworks should be built to streamline the efficient sharing of data and information about the AI decision-making between developers and implementers.

#### Public Benefit

Ensuring that the harms to individual patients and potential harms to disadvantaged groups from risks of the AI-assisted replicating social biases and prejudices is justified with respect to the public benefit of more efficient healthcare systems. Any burdens or risk of harms to groups or individuals from an AI-assisted CDSS should be countered with *significant* public benefits to health systems. The higher the burdens or risk of harm, the greater the benefits there should be (i.e. proportionality). Developers should work closely with implementers to identify and minimise any possible harms to individuals or groups. Any benefits to healthcare systems should not be theoretical but supported with quality evidence that can be evaluated against any real and potential burdens. Where the benefits of these systems do not outweigh the burdens, the systems should not be used or implemented.

### Procedural Values

#### Transparency

AI-assisted CDSS should be able to explain the processes behind its predictions, especially when recommendations made disagree with those of medical professionals. Developers should ensure the AI algorithms used in the CDSS are explainable to the fullest extent possible. The logic processes should be explainable to implementers and programmed in ways that allows the implementer to query each step of the process. Implementers should ensure that if they do not understand the logic behind a recommendation the CDSS has given, that they do not rely on the CDSS.

#### Accountability

Developers of AI-assisted CDSS are responsible for ensuring that recommendations are explainable, while responsibility for any clinical decisions made—irrespective of recommendations made by the CDSS—remains with the medical professional whose patient is under their care.

## Case Study: AI-Assisted CDSS in End-of-life Care

A software developer approaches the head of an intensive care unit (ICU) in a large university hospital to build an AI-assisted CDSS that can predict in real time which patients have or do not have a significant chance of survival to discharge and ability to recover functionally. The Application (‘App’) will not only help tertiary care physicians predict the outcomes for patients admitted to the ICU with higher accuracy but will help hospital administrators better manage scarce resources according to the volume of patients that the system predicts will be admitted to the unit at any one time. Savings in those resources can then be reallocated to another area of the hospital for improved patient care.

## Application of the Deliberative Balancing Approach

### Stating the Problem or Ethical Issue(s)

One of the issues the Head of this ICU considers important is for patients to recover to a functional state rather than receiving invasive treatments, which are very unlikely to benefit the patient in a meaningful way and which also ultimately impact on the Unit’s finite resources. An App that can accurately predict the likelihood of patients surviving the ICU and recovering to a functional state holds promise but the ICU Head weighs up whether this App should be introduced into critical care settings.

Several considerations need to be scrutinised:

1. Healthcare professionals’ primary duties are to benefit and not harm patients. The introduction of this App into ICU would need to be motivated by a desire to ensure that patients are not unnecessarily subjected to invasive treatments that cannot benefit them. Despite the harms associated with the provision of non-beneficial treatments, care would need to be taken that patients are not routinely denied treatment options outright.
2. While cutting unnecessary costs in the ICU is recognised as vital for the functioning of such units, could the introduction of this App be construed as wanting to achieve savings as its primary aim? What impact would that have on the trust the public places in healthcare professionals when patients are in their most vulnerable state?
3. The ICU Head might consider to what extent clinicians could end up relying on the App’s recommendations rather than using their own clinical judgments as well as how their interactions with the App might affect patient care. Conversely, the app can be a useful adjunct to helping clinicians weigh the factors, in light of their professional expertise and experience.
4. She may also be concerned about liability.

The implementer in this case will thus need to weigh up the potential benefits to patients in avoiding highly invasive interventions and more efficient use of ICU resources with the risks of some patients being denied intensive care due to poor predicted outcomes. The management of patient and public perceptions regarding the use of Apps in making decisions about the provision or withholding (or even withdrawal) of treatments would also have to be considered. The advantage of an App that can more accurately predict the outcomes of patients admitted to an ICU is that it may provide clinicians with greater certainty and objective support when formulating prognoses and deciding on the relative benefits and harms associated with invasive and aggressive ICU treatments. These decisions entail many complex evaluations and doctors working in these settings are acutely aware of the highly subjective nature of determining whether patients are likely to survive in the ICU, how much time they may have before death while still in hospital or, if discharged, how much functionality they will have once they leave the ICU.

AI calculations that correspond with the doctors’ assessments can give them greater confidence and a more objective ‘best estimate’ of when certain treatments will have no beneficial outcome for the patient, as a result of the severity of their condition. Communicating this to the patient’s family can help the family avoid forming unrealistic expectations and utilise the remaining time in a meaningful way. In addition to reducing patient harm by not exposing patients to aggressive non-beneficial treatments, increased resource utilisation with minimal beneficial outcome would be curbed and there may also be secondary benefits such as giving hospital administrators more objective information on when beds are likely to become available for incoming patients requiring urgent attention.

Problems may arise where the App predictions *do not* correspond with assessments made by doctors and they are unable to account for how the App has arrived at its recommendation. This problem could be avoided if the App’s logic is available and clearly explainable but that will depend on the developer programming the App well for this purpose and continually monitoring the App as it learns from new data that are being continuously inputted, unless the App is frozen and programmed to not continuously learn.

Potential harms could accrue where patients do not receive the treatments as a result of determinations made solely by the App which they might otherwise have been offered. For example, if the App’s calculation is relied on without further input from clinicians, the determination made by the App could become a self-fulfilling prophecy. Thus, the App must capture the decision-making process of the clinician to prevent biased training data from entering the re-training scheme. Likewise, harms to patients could accrue if treatments were withdrawn without careful consideration of factors that AI cannot account for. Such factors include the values, preferences, and needs of both the patient and their loved ones which should continue to contribute to decisions about the patient even if such Apps are in use. Furthermore, even the most accurate estimations of death come with a margin of error which, in the context of end-of-life decision-making, means that there is always a chance of survival beyond the initial approximate estimations, as research has shown (Berge et al. 2005).

Simply deferring to the App as the sole means of definitively ascertaining the likelihood of survival to discharge as well as imminent death and the suitability of withholding or withdrawing treatments have the potential to erode the trust given to doctors and to the medical profession more generally. In addition, relying on technology alone for such decisions could cause considerable psychological harms to the family, as it may appear that clinicians have abandoned the patient to a machine and, in doing so, objectified the patient and reduced their dignity. There may also be risks that, due to skewed data sets, the App will more frequently predict shorter survival times for certain groups of patients that are more likely to have poorer outcomes due to socially mediated determinants of health. These determinants may arise from socioeconomic circumstances and other inequities that an App may not recognise and inadvertently reinforce if not adequately, carefully, and purposefully accounted for in the algorithm.

### Getting the Facts About the Problem

The facts in this case will rely on the accuracy and programming of the AI algorithm, which should be supported by research before the App is introduced into any CDSS. Data should also be generated on how doctors use the App in their communications with patients to determine how much, if at all, it is likely to support or interfere with the patient-doctor relationship. There is already evidence to suggest that families with unrealistic patient outcome expectations influence excessive utilisation of non-beneficial ICU treatments (Berge et al. 2005). The App may therefore assist clinicians in better communicating their concerns about the patients’ chance of survival and exposure to unnecessary, highly burdensome treatments. Evidence should also be generated on how much more efficiently ICUs run when resources are not allocated to treatments for conditions that cannot be remedied and to patients for whom such interventions bring little to no benefit (and may, in fact, cause harm). The costs incurred when patients are later re-admitted to hospital if discharged prematurely. Prognostic scoring can be compared with those of other institutions.

### Identifying the Relevant Substantive and Procedural Values

The substantive value of *justice* may be considered if evidence emerges of systematic biases being built into the programming of the AI algorithms that prejudice and disadvantage certain social groups. However, the most relevant substantive values to this case are *professional integrity* and *public benefit*. The quality of care that doctors provide to patients and the respect that these professionals demonstrate towards the ICU patient and their family by treating the patient with dignity contributes to their professional integrity. While the family’s values and wishes should be considered and respected, professional integrity requires that doctors do not yield to family demands to implement highly invasive treatments that will not benefit the patient and may cause unnecessary pain or the prolongation of the dying process.

As the costs of healthcare increase, greater efficiency in hospitals is a public good that benefits society more widely. Hospital administrators are thus compelled to implement systems that can reduce costs and make use of scarce resources more efficiently. AI-assisted prognostic scoring systems may assist with both these seemingly conflicting mandates. Such Apps are similar to conventional electronic prognostic scoring, such as APACHE (Niewinski et al. 2014), with an added benefit, amongst others, of saving significant time for clinicians in entering data as this information will be automatically retrieved from the patient’s EHR.^7^ These systems can be helpful in re-enforcing clinical decisions made and providing reassurance to healthcare professionals for these complex and ethically demanding decisions (Berge et al. 2005). This reassurance can also assist when communicating with the patients’ families. However, they should never replace the clinical judgements of doctors or undermine the care given to patients as a result of recommendations the AI algorithms make.

In managing these issues, the procedural values of *transparency* and *accountability* are critical. The developer will need to ensure that the App’s logic is easily understood and explainable to the doctors who will use the App, particularly so they can understand how the AI system has formulated a prognosis when it is inconsistent with their own assessment of a patient surviving an invasive intervention in the ICU. Use of the software should be disclosed to the patient and/or their family in making decisions while acknowledging its limitations in making judgements about values. Doctors should always remain accountable for their decisions, irrespective of whether they are assisted by an AI system or not. While an issue that should be considered, there is currently no evidence that doctors will become so over-reliant on the CDSS that they will disregard their own judgements in deference to the App. This is likely to be especially so in end-of-life decision-making that involves not only clinical factors but moral considerations that AI systems will not account for.

### Considering the Options and Making an Ethical Decision

The options in this case are to either implement the AI-assisted CDSS into the ICU for prognostic scoring or not. The value of implementing this system is to benefit patients and their families by not exposing them to highly invasive interventions unnecessarily and giving them realistic expectations of their survival beyond the ICU. Given the App’s purpose of *facilitating* decision-making, its use in this highly demanding and morally challenging clinical context would be of public benefit. The public would have even greater confidence that decisions made in the complex ICU setting were carefully considered by experts and supported by advanced technology.

The App may allow the hospital to operate the ICU more efficiently and save money but this consideration should be secondary to improved patient care. If the App was adopted solely or even primarily for the purpose of saving money, patients and their families would be justified in having concerns that the patient’s worth is being judged. They would also justifiably question the professional integrity of the doctors involved. However, if the primary aim was to avoid imposing significant burdens and harms on patients through the provision of non-beneficial treatments, the moral value of such efforts would be justified, as the patient would be at the centre of such considerations. It would be more acceptable then to also view the non-use of non-beneficial treatments as a benefit for other patients who may require them thus ultimately providing benefits to the broader public. In cases where use of the App resulted in a decision which conflicted with the health professional’s, it would be imperative that the clinical team responsible for the patient could understand the basis for its decision to ensure that the technology provided maximum transparency.

## Conclusion

Given the rising costs of healthcare, the continued development and implementation of AI-assistance into clinical decision-making is a likely inevitability. This domain has highlighted some of the ethical issues that may arise with the implementation of these systems and the relevant values that decision-makers can draw on in the design and implementation of AI-assisted CDSS into practice. Specifically, the values of professional integrity and accountability will play most prominently at the level of patient care, while values of justice and the potential for group harms must be balanced against the imperatives for public benefit at the societal level. The value of transparency cuts across both with trust in the medical profession and healthcare systems at stake.

## Footnotes

1 For more information about the machine learning of AI analytics, see the Information Commissioner’s Office (2017)

2 Hard-coded software is traditional software that operates based on fixed rules that are coded by programmers.

3 For example, anti-discrimination laws in Australia have not prevented discriminatory practices of insurers based on genetic test results (Tiller et al. 2019)

4 See for example the General Data Protection Regulation in the EU, Articles 6 and 9.

5 A broader issue here is whether aggregated data is in fact truly anonymised, a question that is beyond the scope of this work.

6 See also the work of the Ethics Committee of the American College of Epidemiology (Salerno et al. 2017) and Kenneth Goodman (2015).

7 They are also beneficial in that conventional scoring systems are programmed from the outset and remain static until new data is available to revalidate the score, while AI systems are not hard-coded and can be incrementally trained to improve its accuracy.

## References

[CR1] Berge KH, Maiers DR, Schreiner DP, Jewell SM, Bechtle PS, Schroeder DR, Stevens SR, Lanier WL (2005). Resource Utilization and Outcome in Gravely Ill Intensive Care Unit Patients with Predicted In-Hospital Mortality Rates of 95% or Higher by APACHE III Scores: the Relationship with Physician and Family Expectations. Mayo Clinic Proceedings.

[CR2] Burrell, Jenna. 2016. How the Machine ‘Thinks’: Understanding Opacity in Machine Learning Algorithms. *Big Data & Society* 3 (1): 1–12. 10.1177/2053951715622512.

[CR3] Dagliati A, Marini S, Sacchi L, Cogni G, Teliti M, Tibollo V, De Cata P, Chiovato L, Bellazzi R (2018). Machine Learning Methods to Predict Diabetes Complications. Journal of Diabetes Science and Technology.

[CR4] Dagliati A, Tibollo V, Sacchi L, Malovini A, Limongelli I, Gabetta M, Napolitano C (2018). Big Data as a Driver for Clinical Decision Support Systems: A Learning Health Systems Perspective. Frontiers in Digital Humanities.

[CR5] El-Sappagh, Shaker, and Mohammed Elmogy. 2016. A Decision Support System for Diabetes Mellitus Management. *Diabetes Case Reports* 1:102. 10.4172/2572-5629.1000102.

[CR6] Goodman KW, Berner ES (2007). Ethical and Legal Issues in Decision Support. *Clinical Decision Support Systems: Theory and Practice*.

[CR7] Goodman KW (2010). Ethics, Information Technology, and Public Health: New Challenges for the Clinician-Patient Relationship. The Journal of Law, Medicine & Ethics.

[CR8] Goodman KW (2015). Ethics, Medicine, and Information Technology: Intelligent Machines and the Transformation of Health Care.

[CR9] Guo E, Jacobs DB, Kesselheim AS (2017). Eliminating Coverage Discrimination Through the Essential Health Benefit's Anti-Discrimination Provisions. American Journal of Public Health.

[CR10] Information Commissioner’s Office. 2017. Big Data, Artificial Intelligence, Machine Learning and Data Protection. https://ico.org.uk/media/for-organisations/documents/2013559/big-data-ai-ml-and-data-protection.pdf. Accessed 19 Feb 2019.

[CR11] Institute of Medicine. 2007. The Learning Healthcare System. https://www.ncbi.nlm.nih.gov/books/NBK53494/. Accessed 9 Feb 2019.

[CR12] Jiang F, Jiang Y, Zhi H, Dong Y, Li H, Ma S, Wang Y, Dong Q, Shen H, Wang Y (2017). Artificial Intelligence in Healthcare: Past, Present and Future. Stroke and Vascular Neurology.

[CR13] Kass NE, Faden RR, Goodman SN, Pronovost P, Tunis S, Beauchamp TL (2013). The Research-Treatment Distinction: A Problematic Approach for Determining Which Activities Should Have Ethical Oversight. Hastings Center Report.

[CR14] Lim HY (2017). Data Protection in the Practical Context – Strategies and Techniques.

[CR15] McGibbon E, Etowa J, McPherson C (2008). Health-Care Access as a Social Determinant of Health. The Canadian Nurse.

[CR16] National Academies of Science, Engineering, and Medicine. 2017. *Communities in Action: Pathways to Health Equity*. Washington DC: The National Academies Press. 10.17226/24624. 28418632

[CR17] Niewinski G, Starczewska M, Kanski A (2014). Prognostic Scoring Systems for Mortality in Intensive Care Units--the APACHE Model. Anaesthesiol Intensive Therapy.

[CR18] Rosenbaum S (2009). Insurance Discrimination on the Basis of Health Status: An Overview of Discrimination Practices, Federal Law, and Federal Reform Options. The Journal of Law, Medicine & Ethics.

[CR19] Salerno, Jennifer H., Bartha M. Knoppers, Lisa M. Lee, Wayway M. Hlaing, and Kenneth W. Goodman. 2017. Ethics, Big Data and Computing in Epidemiology and Public Health. *Annals of Epidemiology* 27 (5): 297–301. 10.1016/j.annepidem.2017.05.002. 10.1016/j.annepidem.2017.05.002 28595734

[CR20] Tiller, Jane, Susan Morris, Toni Rice, Krystal Barter, Moeen Riaz, Louise Keogh, Martin B. Delatycki, Margaret Otlowski, and Paul Lacaze. 2019. Genetic Discrimination by Australian Insurance Companies: a Survey of Consumer Experiences. *European Journal of Human Genetics*. 10.1038/s41431-019-0426-1. 10.1038/s41431-019-0426-1 PMC6906286 31281182

[CR21] Wagholikar KB, Sundararajan V, Deshpande AW (2012). Modeling Paradigms for Medical Diagnostic Decision Support: a Survey and Future Directions. Journal of Medical Systems.

[CR22] Yu PP (2015). Knowledge Bases, Clinical Decision Support Systems, and Rapid Learning in Oncology. Journal of Oncology Practice.

[CR23] Xafis, Vicki, G. Owen Schaefer, Markus K. Labude, Iain Brassington, Angela Ballantyne, Hannah Yeefen Lim, Wendy Lipworth, Tamra Lysaght, Cameron Stewart, Shirley Hsiao-Li Sun, Graeme T. Laurie, and E. Shyong Tai. 2019. An Ethics Framework for Big Data in Health and Research. *Asian Bioethics Review* 11 (3). 10.1007/s41649-019-00099-x. 10.1007/s41649-019-00099-x PMC7747261 33717314

